# Myopic Loss Aversion under Ambiguity and Gender Effects

**DOI:** 10.1371/journal.pone.0161477

**Published:** 2016-12-14

**Authors:** Iñigo Iturbe-Ormaetxe, Giovanni Ponti, Josefa Tomás

**Affiliations:** 1 Departamento de Fundamentos del Análisis Económico, Universidad de Alicante, 03071 Alicante, Spain; 2 Department of Economics, University of Chicago, 1126 East 59th Street, Chicago, IL 60637, United States of America; 3 Dipartimento di Economia e Finanza, LUISS Guido Carli, viale Romania 32, 00197, Roma, Italy; University of the Basque Country, SPAIN

## Abstract

Experimental evidence suggests that the frequency with which individuals get feedback information on their investments has an effect on their risk-taking behavior. In particular, when they are given information sufficiently often, they take less risks compared with a situation in which they are informed less frequently. We find that this result still holds when subjects do not know the probabilities of the lotteries they are betting upon. We also detect significant gender effects, in that the frequency with which information is disclosed mostly affects male betting behavior, and that males become more risk-seeking after experiencing a loss.

## Introduction

The so called *equity premium* is the difference between the return on stocks and the return on risk-free assets. Mehra and Prescott [1] claim that this difference is too large to be explained by standard economic models, and coin the term “equity premium puzzle” to refer to this anomaly. They show that, to rationalize this phenomenon, individuals should hold a degree of risk aversion implausibly high. Benartzi and Thaler [2] suggest that *Myopic Loss Aversion* (MLA) may help to solve this puzzle. MLA is described by two features, *i*) loss aversion and *ii*) mental accounting. Loss aversion is a cornerstone of Prospect Theory (Kahneman and Tversky [3]) and refers to the tendency of individuals to weigh losses more heavily than gains. Mental accounting refers to the process individuals use to code and evaluate economic outcomes. In this respect, one relevant aspect is the frequency with which individuals evaluate the performance of their investments. Precisely, MLA refers to how investments are evaluated over time (frequency dimension) and cross-sectionally (whether individuals evaluate each stock in isolation, or evaluate the performance of their entire portfolio). Benartzi and Thaler [2] find that, under MLA, individuals who evaluate their investments less frequently are more willing to take risks.

Several authors have designed experiments to test the empirical content of MLA in the lab. Thaler *et al.* [4] design a portfolio choice experiment and find evidence that subjects are, indeed, loss averse and that risk-taking behavior increases when information is released less frequently. Thaler *et al.* [4] refer to this process as a way of “reducing myopia”. Of most interest to us is the paper by Gneezy and Potters ([5], GP97 hereafter), who set up an experimental “investment game” in which subjects face a sequence of nine i.i.d. lotteries. Each one of these lotteries gives a probability of 1/3 of winning 2.5 times the amount invested, and a probability of 2/3 of losing it. In one treatment (“high frequency”, HF) subjects play the nine rounds one by one. At the beginning of each round they have to choose how much to invest. Then, before proceeding to the next round, they are informed about the realization of the lottery. In the other treatment (“low frequency”, LF) subjects play rounds *in blocks of three*. That is, they must invest the same amount for the three lotteries in each block. These decisions are taken at the beginning of rounds 1, 4, and 7. Subjects are informed about the realization of the lotteries in each block at the end of rounds 3, 6, and 9. This design feature has an important impact on risk-taking behavior: consistently with MLA, *subjects invest significantly more in the LF treatment*. Other scholars have replicated GP97 experimental setting finding consistent evidence: people invest more when their myopia is corrected ([6, 7, 8, 9]).

A common feature of this literature is that investment decisions are taken *under risk*, since subjects know the probabilities of the lotteries they are betting upon. This is only a first approximation of most real-life situations, where it is almost impossible to know precisely the probabilities associated with future returns when buying stocks, or choosing a job. In this respect, our main goal is to check whether GP97’s findings *are robust to ambiguity*, i.e., if they carry over to situations in which subjects are unaware of the winning probability. To this aim, we borrow the basic GP97 layout, replicate their two treatments and add two additional treatments (one with HF, another with LF) in which subjects are not informed about the winning probability. In this latter condition, consistently with Ellsberg’s [10] well known “paradox”, we expect subjects to bet less than in the corresponding treatments under risk.

Consistently with our working hypothesis, our experimental evidence shows that GP97’s findings *are reinforced by the presence of ambiguity*, in that the increase in risk-taking from the HF to the LF treatment is even higher under ambiguity, as the average investment increases by 38.9% from HF to LF under risk, and by 57.1% under ambiguity. We also detect some evidence of ambiguity aversion (in that subjects, on average, invest less than in the corresponding risky conditions). This “ambiguity effect”, however, seems less prominent than the “frequency effect”. In this respect, our results are in line with those of Charness and Gneezy [11], who also compare, similar to ours, evidence on investment games under risk (Treatment 5) and ambiguity (Treatment 6) and find that ambiguity only marginally affects average investment levels. However, in both treatments, *individuals play only once*, i.e., they do not compare HF with LF.

We also look at gender differences in our data. In this respect, we find that females invest, on average, less than males, 46.6% and 53.5% of their endowment, respectively. We get these figures when we pool together all four treatments. Interestingly enough, this difference is entirely due to the two LF treatments: 53.3% (females) *vs.* 65.6% (males). By contrast, in the two HF treatments average investments are practically identical: 40.4% (females) *vs.* 40.3% (males). Another interesting finding is that the increase in risk-taking from HF to LF treatments that we observe is mainly due to *changes in male behavior*. The increase from the HF to the LF treatments is 62.8% for males, and 31.9% for females. Finally, we also find that, in our sample, males are much more affected by a previous loss. Specifically, we find that subjects are more risk-seeking after experiencing a loss, increasing their bets by 15.4%, on average. However, while females only increase their investment by 11.6%, males increase theirs by 19.4%.

## Materials and Methods

The experimental protocol reported in this paper was approved by the Ethics Committee at the Faculty of Economics and Business at the Universidad de Alicante. All subjects signed written informed consent prior to participation in the study.

### Sessions

We ran 4 sessions of 24 subjects, for a total of 96 participants, 46 male and 50 female, recruited from the undergraduate population of the Bachelor Degree in Business Administration of the Universidad of Alicante. Experiments were carried out with paper and pencil in classrooms which seat 100 individuals, so that the 24 participants in each session could be easily separated from one another and decide in isolation.

We borrow our control treatments from GP97, who set up an experiment in which subjects face a sequence of nine independent lotteries. In each round, subjects are endowed with 100 euro cents. They have to decide the amount *x* ∈ [0, 100] of their endowment they want to invest in a lottery that returns two and a half times the amount invested with a probability of 1/3 while, with a probability of 2/3, all the invested money is lost. Subjects were privately paid off in cash their cumulative earnings right after the end of the experiment. Average earnings were 9.36 euro for an experiment that, on average, lasted 40 minutes.

### Treatments

In the first treatment, T1 (“high frequency”, HF), subjects play the nine rounds one by one. At the beginning of each round they have to choose how much to invest. Then, before proceeding to the next round, they are informed about the realization of the lottery. In the second treatment, T2 (“low frequency”, LF) subjects play rounds *in blocks of three*. That is, they must invest the same amount for the three lotteries in each block. These decisions are taken at the beginning of rounds 1, 4, and 7. Subjects are informed of the realization of lotteries at the end of rounds 3, 6, and 9. Our two baseline treatments, T1 and T2, replicate GP97 conditions under risk.

Our alternative treatments, T3 and T4, implement under ambiguity the HF and LF conditions, respectively. The winning probability, *p* = 1/3, is the same as in treatments T1 and T2, but no information is disclosed about it, except that it stays constant across rounds. We refer to T1 and T2 (T3 and T4) as the *Risky* (*Ambigous*) treatments, respectively, as sketched in Table 1.

**Table 1:** The four treatments.

Treatments	Risk	Ambiguity
High Frequency	T1 (HF-R)	T3 (HF-A)
Low Frequency	T2 (LF-R)	T4 (LF-A)

## Results

**Fig 1:**
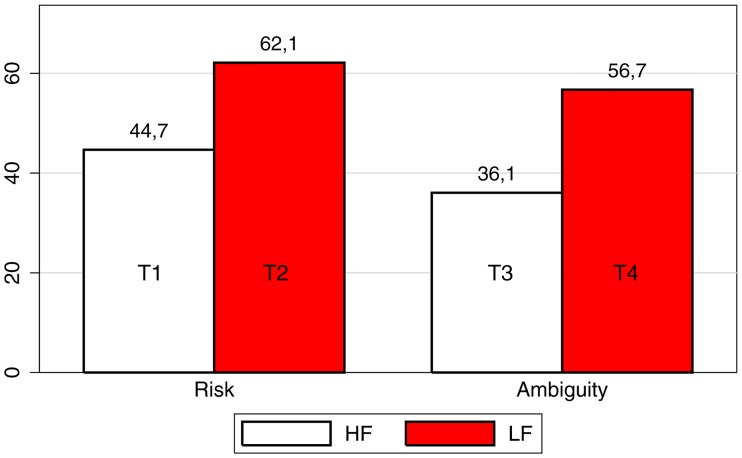
Average investment by treatment.

Fig 1 tracks average investments in the four treatments. As Fig 1 shows, our results are in line with the literature we discussed in the introduction: *subjects invest more in the LF treatments*. This finding is consistent with all other replications of GP97 we are aware of (take, e.g., [6, 7]): people invest more when their myopia is corrected.

As for our Ambiguity treatments, Fig 1 shows that the difference in behavior between HF and LF sessions (T3 *vs* T4) is even higher. Under risk, average investment increases by 39%; under ambiguity, it increases by 57%. Differences between HF and LF are, in both cases, highly significant by standard non parametric Mann-Whitney statistics (*p* < 0.001).

Moreover, Fig 1 also shows *some evidence of ambiguity aversion* in that, keeping the frequency constant, subjects invest less in the ambiguous treatments. In the HF (LF) case, average investment falls by 19.2% (8.7%), respectively. These differences, however, are not statistically significant (*p* = 0.322 and *p* = 0.227).

We also conduct a one-way between-subject ANOVA to test whether average investments are significantly different across treatments. The null hypothesis in ANOVA is that all mean investment is the same across treatments. We reject the null at the *p* < 0.001 level [*F*(3,92) = 9.24, *p* = 0.0000]. Post hoc comparisons using the Tukey HSD (Honestly Significant Difference) test indicate that differences are statistically significant at the 5% level between T1 and T2, between T2 and T3, and between T3 and T4. This confirms that subjects invest more in the LF than in the HF treatments.

We present additional evidence in Table 2, where we decompose our data in four panels. Panel 2a replicates Table 1 in GP97 using our data. Panel 2b compares HF and LF treatments under ambiguity. Panel 2c compares risk and ambiguity for the HF treatments, while Panel 2d does the same for the LF treatments.

**Table 2:** Average investment by treatment.

	T1	T2	M-W	T3	T4	M-W
Rounds 1–3	42.5 (22.8)	51.9 (19.0)	-1.71 [.088]	31.6 (20.3)	49.7 (20.2)	-3.42 [.001]
Rounds 4–6	42.4 (25.4)	62.4 (21.2)	-3.12 [.002]	33.4 (21.2)	52.9 (17.7)	-3.16 [.002]
Rounds 7–9	49.0 (27.0)	72.2 (26.4)	-2.93 [.003]	43.1 (29.7)	67.7 (19.3)	-2.98 [.003]
All rounds	44.7 (24.9)	62.1 (23.6)	-2.84 [.004]	36.1 (24.3)	56.7 (20.4)	-3.83 [.000]
	(2a)			(2b)		
	T1	T3	M-W	T2	T4	M-W
Rounds 1–3	42.5 (22.8)	31.6 (20.3)	1.82 [.069]	51.9 (19.0)	49.7 (20.2)	.39 [.699]
Rounds 4–6	42.4 (25.4)	33.4 (21.2)	1.17 [.243]	62.4 (21.2)	52.9 (17.7)	1.31 [.191]
Rounds 7–9	49.0 (27.0)	43.1 (29.7)	.60 [.549]	72.2 (26.4)	67.7 (19.3)	.84 [.400]
All rounds	44.7 (24.9)	36.1 (24.3)	.99 [.322]	62.1 (23.6)	56.7 (20.4)	1.21 [.227]
	(2c)			(2d)		

Number of individuals in round brackets. Standard deviations in square brackets.

As Panel 2a shows, our results are very much in line with those of GP97 and Haigh and List [6], in that we find that average investment is significantly higher in T2, thus confirming the results obtained by our ANOVA tests. Differences are always statistically significant, whether we consider each block of three rounds alone, or all rounds altogether. In Tables 2–4 statistical significance is measured by (2-tailed) non-parametric Mann-Whitney (M-W) non-parametric statistics.

**Table 3:** Treatment effects by gender.

	T1	T2	M-W	T3	T4	M-W
males	44.8 (9)	71.2 (11)	-2.70 [.007]	37.2 (13)	60.8 (13)	-3.08 [.002]
females	44.6 (15)	54.5 (13)	-1.27 [.205]	34.7 (11)	52.0 (11)	-2.37 [.018]
All	44.7 (24)	62.1 (24)	-2.84 [.004]	36.1 (24)	56.7 (24)	-3.83 [.000]
	T1	T3	M-W	T2	T4	M-W
males	44.8 (9)	37.2 (13)	.63 [.526]	71.2 (11)	60.8 (13)	1.71 [.086]
females	44.6 (15)	34.7 (11)	.75 [.451]	54.5 (13)	52.0 (11)	.14 [.885]
All	44.7 (24)	36.1 (24)	.99 [.322]	62.1 (24)	56.7 (24)	1.21 [.227]

Number of individuals in round brackets. Standard deviations in square brackets.

**Table 4:** Marginal effects.

Variables	Model 1	Model 2	Model 3
**LF**	21.319***(5.000)	17.455***(5.081)	19.220***(5.700)
**Ambig**	−12.848**(5.338)	−12.828**(5.288)	−12.722**(5.837)
**Female**	−4.777 (5.352)	−4.722 (5.303)	−4.668 (5.804)
**Profit**	0.019***(0.004)	0.010** (0.005)	0.010* (0.006)
**Lastperiod**		13.849***(4.013)	15.092***(4.349)
**Loser**			7.748**(3.388)
**Observations**	576	576	480
**Log likelihood**	−2300.306	−2290.553	−1870.808

Standard errors in round brackets;

*, **, *** indicate significance levels of 90, 95 and 99%, respectively.

When we move to our “ambiguous” treatments, T3 and T4, we observe -again- that subjects invest significantly more in the LF treatment. In fact, differences between T3 and T4 are slightly bigger than differences between T1 and T2.

Our experimental evidence shows that GP97’s findings *are reinforced by the presence of ambiguity*, in that the increase in risk-taking from the HF to the LF treatment is even higher, as the average invest increases from 44.7% in HF to 62.1% in LF under risk, and from 36.1% in HF to 56.7% in LF under ambiguity. Contrary to what has been suggested by Blavatskyy and Pogrebna [12, 13], we find that the difference between LF and HF treatments is not driven by subjects with “extreme” behavior, i.e., who systematically invest either 0% or a 100% of their endowments. Differences remain significant even when we exclude such individuals from our sample.

When we look at Panel 2c, we conclude that subjects’ behavior exhibits some ambiguity aversion, in that the average investment is lower in T3. However, the difference in average investment between T1 and T3 is not statistically significant, a result that we also obtain when we repeat the same exercise using data from T2 and T4 (LF treatments).

Results up to now seem to point out that, in our experiment, the “ambiguity effect” is lower than the “frequency effect”. That is, subjects invest more when myopia is corrected, independently on whether they know the winning probability. By contrast, keeping the choice frequency constant, subjects’ investment, either under risk, or ambiguity, is practically the same. This is consistent with the evidence reported in Charness and Gneezy [11].

### Gender effects

**Fig 2:**
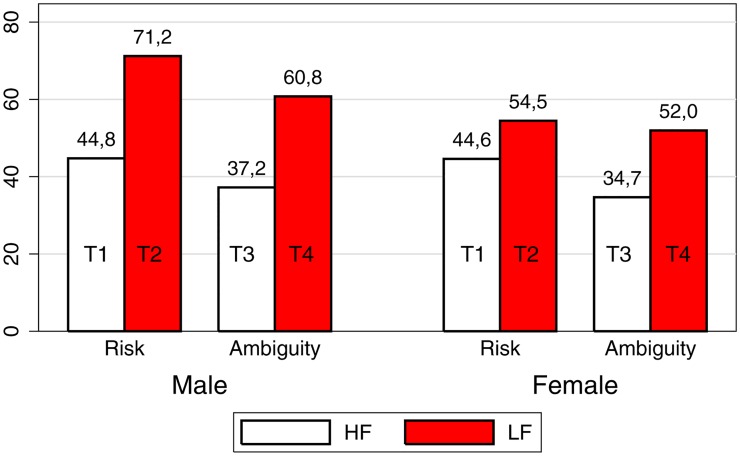
Average investment by treatment and gender.

In the previous section we have seen that individuals invest more in the LF than in the HF treatment, both under risk and ambiguity. In this section we want to see whether this “frequency effect” has a gender component. Fig 2 tracks average investment disaggregated by gender in the four treatments. We find that, although females invest less than males in all treatments, this difference is only significant in T2 (*p* = 0.058). This result differs slightly from previous works in the literature. Croson and Gneezy [14] report that females are, on average, more risk averse than males. However Nelson [15] finds more mixed results. She finds that some studies report higher average female risk taking and others where the gender difference lacks statistical significance. This is also the conclusion obtained by Filippin and Crosetto [16]. Charness and Gneezy [17] gather results from ten experiments similar to our baseline treatment (T1) and conclude that, on average, males tend to take higher risks than females. However, only two out of the ten studies in Table 4 of Charness and Gneezy [17] can be directly compared to ours: Haigh and List [6] and Bellemare *et al.* [7]. Looking at the numbers corresponding to these two studies, it seems that gender differences are small: in Haigh and List [6] the average bet is 58.3 for males vs. 55.6 for females, while in Bellemare *et al.* [7] the average bet is 45.5 for males vs. 42.7 for females. In this respect, it seems that our results are not so different from theirs. We can also compare the five studies in Charness and Gneezy [17] in which individuals play only once with our experiment by looking at what happens in our first round (or in our last round). In period 1 of the baseline treatment, average bet by males is 48.9 while average bet by females is 40. In period 9 of the baseline treatment, average bet by males is 59.9 while average bet by females is 48.9.

Looking at Fig 2 we also see that male behavior displays a higher variability across treatments. We run, again, some ANOVA tests, separately by gender. The ANOVA test restricted to males rejects, once again, the null hypothesis that all mean investment is the same across treatments [*F*(3,42) = 9.54, *p* = 0.0001, *N* = 46]. By contrast, when we run the same test for females, we can only reject the null at the 10% confidence level [*F*(3,46) = 2.22, *p* = 0.0981, *N* = 50]. This result is remarkable, because it points out that *differences in behavior across treatments are mainly driven by males.*

As we already noticed, the frequency dimension seems to have a stronger effect than the ambiguity dimension at the aggregate level. With this premise, Table 3 measures treatment effects disaggregated by gender.

Table 3 confirms the preliminary evidence of Fig 2: the difference in average investment between T1 and T2 *is mainly driven by males.* In fact, although females invest more in T2 than in T1 (54.5 vs. 44.6), this difference is not statistically significant. Then, we see that the “frequency effect” we found above is mainly driven by the large difference in average investment by males from T1 to T2 (44.8 vs. 71.2). Thaler *et al.* [4] interpret the change in framework from T1 to T2 as a “correction of myopia.” This is done in two ways: (i) by committing individuals for multiple periods, and (ii) by giving them information relatively infrequently. With this interpretation in mind, our results seem to imply that, under risky conditions, female risk-taking behavior is less sensitive to a correction of myopia. Regarding T3 and T4, we find that both genders invest significantly more in the LF treatment, although the effect is, again, stronger for males. In the lower panel of Table 3 we compare average investment under risk and ambiguity. Both males and females invest less under ambiguity, but the only statistically significant difference is that for males in the LF treatments, that is, when we compare T2 with T4.

### Individual behavior

This section aims at identifying the main factors affecting subjects’ decisions on how much to invest, a variable that takes values from 0 to 100. In treatments T1 and T3 subjects take 9 decisions while, in treatments T2 and T4, they only decide three times. Therefore, the total number of observations is 24×2×9+24×2×3 = 576. Since our dependent variable is censored between 0 and 100, we estimate a random effect tobit model, which assumes that there is a latent variable $y_{it}^{*}=x_{it}^{\prime }\beta +u_{i}+\epsilon_{it}$, where $u_{i}∼N\left(0,\sigma_{u}^{2}\right)$ is the individual random effect and $\epsilon_{it}∼N\left(0,\sigma_{\epsilon }^{2}\right)$ is an idiosyncratic error term. The observable variable *y*_*it*_ (in our case, the amount invested in period *t*) is defined as:

$$y_{it}=\{\begin{array}{ll} 0 & \text{if }y_{it}^{*}\leq 0\\ y_{it}^{*} & \text{if }y_{it}^{*}\in \left(0,100\right)\\ 100 & \text{if }y_{it}^{*}\geq 100. \end{array}$$ (1)

In our regressions, we consider the following explanatory variables:

- **LF**. A binary index, positive for the LF treatments, T2 and T4.
- **Ambig**. Another binary index, positive for the ambiguous treatments, T3 and T4.
- **Female**. Gender dummy, positive for female.
- **Lastperiod**. Time dummy, positive for period 9 (HF treatments) and period 7 (LF treatments), respectively. Thaler and Johnson [18] defend that outcomes that offer the chance to break-even are especially attractive. This “break-even effect” could explain why subjects invest more in their last round.
- **Loser**. Takes value 1 when the individual experienced a loss in the previous round (treatments T1 and T3) or when she experienced a loss in at least two in three rounds in the previous block (treatments T2 and T4). In previous studies it has been found that subjects modify their behaviour after experiencing a loss. Hopfensitz [19] performs a meta study using data of five experiments, including GP97, and finds that previous outcomes influence investing behavior, namely, *small gains reduce investment and small losses increase investment*.
- **Profit**: This variable takes, in period *t*, the sum of cumulative profits up to period *t* − 1.

Tables 4 and 5 report the marginal effects of our regressors. As a robustness check, we also report in S1 Appendix the estimates of the standard to bit model without random effects (Table A in S1 Appendix), and a random-effect uncensored regression, excluding the extreme observations (Table B in S1 Appendix), along the lines of the critique by Blavatskyy and Pogrebna [13]. We also include in the regressions interaction terms. S1 Table reports all the estimated coefficients.

**Table 5:** Marginal effects by gender (Model 3).

Variables	Male	Female	All
**LF**	25.150***(8.280)	14.030*(7.825)	19.220***(5.700)
**Ambig**	−12.510 (8.548)	−12.910 (7.965)	−12.722**(5.837)
**Lastperiod**	23.940***(5.873)	7.353 (5.467)	15.092***(4.349)
**Loser**	10.400**(4.842)	5.427 (4.401)	7.748**(3.388)

Standard errors in round brackets;

*, **, *** indicate significance levels of 90, 95 and 99%, respectively.

The estimates of Table 4 are based on three alternative models. In Model 1 we include **LF**, **Ambig**, and **Female** together with all their interactions, and **Profit**.

For the purpose of illustration we write down Model 1 explicitly:

$$\begin{array}{ccc} x_{i}^{\prime }\beta & = & \beta_{0}+\beta_{LF}LF_{i}+\beta_{UN}Ambig_{i}+\beta_{G}Gender_{i}+\beta_{LU}LF_{i}Ambig_{i}+\beta_{LG}LF_{i}Gender_{i}+\\ & & +\beta_{UG}Ambig_{i}Gender_{i}+\beta_{LUG}LF_{i}Ambig_{i}Gender_{i}+\beta_{P}\mathrm{Pr}ofit_{i} \end{array}$$

This means that our estimation includes seven dummies, one continuous variable, and a constant. In Model 2 we also include *Lastperiod*, which we interact with all variables in Model 1 except **Profit**. Model 2 comprises fourteen dummies and a constant. We decide to interact at most three variables to avoid running into identification issues. In fact, for some of these interaction, there is no variation. Finally, in Model 3 we include the dummy variable **Loser**, and we also include ten additional dummies that capture interactions with all dummies of Model 2. In Model 3 we lose 96 observations because, when we introduce **Loser**, we have to eliminate each individual’s first decision. This implies the elimination of 24×4 = 96 observations.

In all our three models we find that the coefficient of **LF** is positive and highly significant. In Model 3 individuals increase their investment, on average, a 19.2% when they move from T1 to the LF treatments. Our **Ambig** dummy has a negative effect on investment. This variable captures the effect of going from T1 to the treatments with unknown probability. We find a significant effect at the 1% level in all models. In Model 3 we find that, in the Ambiguity treatments, subjects reduce their investment by 12.7%, on average. These results reinforce our claim that the frequency dimension matters more than the ambiguity dimension.

In line with the descriptive statistics of the previous section, in all three models, our gender dummy **Female** is not significant. Interestingly, we also find that, although the variable **Profit** has a positive impact on individual investment, its effect loses significance as we move from Model 1 to Model 3, where we include the other two time-dependent covariates, **Lastperiod** and **Loser**. This seems to indicate that the large increase in investment in the last period is not due to an income effect, but simply to a pure “end-game” effect.

In Models 2 and 3 we find that **Lastperiod** has a strong positive effect, since individuals increase their investment by 15%, approximately. This end-game effect can be due to different reasons. It could be due to a pure end-game effect or to a break-even effect, as we commented above.

In Model 3 we control also for the effect of having experienced a loss in previous rounds. In this respect, the marginal effect of the variable **Loser** is positive and significant: other things equal, individuals increase their investment by 7.7% after experiencing a loss. This effect is comparable to the one obtained by Hopfensitz [19]. Again, a “break-even” effect could explain this behavior. It seems some individuals are trying to recover money lost in previous periods by taking higher risks. Another possible explanation could be a version of the “gambler’s fallacy” ([20]). Some individuals may believe that the probability of winning increases after having lost. Finally, also Prospect Theory predicts that in the region of losses individuals become more risk seekers if they do not integrate losses. In our experiment we cannot disentangle which one of these different explanations (or which combination of them) is driving the result.

Now we want to calculate the marginal effects of **LF**, **Ambig**, **Lastperiod** and **Loser** by gender. The idea is to check whether our regressors have *gender-specific component*, in line with our previous results. We do it only for Model 3, whose marginal effects by gender are reported in Table 5.

To explain Table 5, let us focus, for instance, on the first entry (25.15%). This number represents the effect of the dummy **LF** on male subjects only. On average, males increase their investment by 25% when they move from T1 to the low frequency treatments. Females also increase their investment, but only by 14%.

It is worth looking at the variables **Lastperiod** and **Loser**. For both variables, we see that the positive effect we found in Table 4 can be entirely attributed to male behavior. With respect to **Lastperiod**, we cannot reject the null hypothesis that females invest in the last decision the same amount as in previous periods. In contrast, males seem to suffer a much stronger end-game effect since, in their last decision, they increase their investment by 23.9%. With respect to **Loser**, again, we cannot reject the null hypothesis that females are not affected by previous losses. By contrast, males have a strong reaction to previous losses, since they raise their investment by 10.4%. From our definition of **Loser**, we also find that males who have won in the previous period tend to reduce their investment. This seems to imply that males are more prone than females to suffer from some of the biases mentioned above, such as the “gambler’s fallacy”, “break-even effects”, etc…

## Discussion

Following GP97, there is a substantial evidence indicating that subjects tend to invest significantly more in the LF treatment than in the HF treatment. In this respect, our experimental evidence shows that this result can be extended to situations in which individuals decide under ambiguity, although we have mixed results on the empirical content of an “ambiguity effect” in investment game experiments.

We also find that investing behavior has a clear gender component, not much at the aggregate level but, rather, when we look at differences in behavior induced by varying the treatment conditions. In this respect, male behavior seems to be much more sensitive to changes in the lottery environment. For example, we find that, when the probability is known, most of the increase in bets when we move from the HF to the LF treatment is due to a change in male behavior. By contrast, we do not find any significant gender difference in how individuals react to the introduction of ambiguity. In this respect, our results are much in line with those that find significant gender differences with respect to specific experimental conditions -take, for instance, the availability of a safe option, as in Filippin and Crosetto [16]- rather than a gender effect which is independent of environmental conditions.

## Supporting Information

S1 Appendix Further Statistical Evidence. (PDF)

S2 Appendix Experimental Instructions. (PDF)

S1 Table Random-effect tobit regressions. (PDF)
