# Supplementary material for: Myopic Loss Aversion under Ambiguity and Gender Effects
Source: PLoS One. 2016 Dec 14;11(12):e0161477. doi: 10.1371/journal.pone.0161477 (PMC5156393; doi:10.1371/journal.pone.0161477)
Supplement: S1 Appendix — (PDF) [file pone.0161477.s001.pdf]

Myopic Loss Aversion under Ambiguity and Gender Effects. S1 Appendix: Further Statistical Evidence

Iñigo Iturbe-Ormaetxe [1], Giovanni Ponti[1,2,3,*], Josefa Tomás[1]

1. Departamento de Fundamentos del Análisis Económico, Universidad de Alicante, 03071 Alicante, Spain.
2. Department of Economics, University of Chicago, 1126 East 59th Street, Chicago IL 60637, United States.
3. Dipartimento di Economia e Finanza, LUISS Guido Carli, viale Romania 32, 00197, Roma, Italy.

* Corresponding Author. E-mail: giuba@ua.es.

Tab A: Standard tobit marginal effects

Variables	Model 1	Model 2	Model 3
**LF**	18.205***(4.156)	14.728***(4.420)	15.724***(4.114)
**Ambig**	−9.901**(4.921)	−9.873**(4.907)	−8.492*(5.138)
**Female**	−3.554 (4.906)	−3.454 (4.866)	−3.709 (5.011)
**Profit**	0.013**(0.006)	0.006 (0.007)	0.007 (0.009)
**Lastperiod**		12.622***(5.126)	11.720**(5.241)
**Loser**			9.575***(3.161)
**Observations**	576	576	480
**Log likelihood**	−2373.112	−2365.808	−1931.751

Standard errors in round brackets; *, **, *** indicate significance levels of 90, 95 and 99%, respectively.

Tab B: RE regressions excluding extreme behavior

Variables	Model 1	Model 2	Model 3
**LF**	20.156***(2.785)	18.344***(2.818)	20.688***(3.156)
**Ambig**	−1.242(2.799)	−1.311(2.843)	−1.643(3.224)
**Female**	−3.990 (2.728)	−4.282 (2.779)	−4.159 (3.168)
**Profit**	0.010***(0.002)	0.006**(0.003)	0.008**(0.004)
**Lastperiod**		6.323**(2.646)	7.045**(2.992)
**Loser**			0.838(1.910)
**Observations**	576	576	480
**Log likelihood**	−2373.112	−2365.808	−1931.751

Standard errors in round brackets; *, **, *** indicate significance levels of 90, 95 and 99%, respectively.
