# Supplementary material for: Myopic Loss Aversion under Ambiguity and Gender Effects
Source: PLoS One. 2016 Dec 14;11(12):e0161477. doi: 10.1371/journal.pone.0161477 (PMC5156393; doi:10.1371/journal.pone.0161477)
Supplement: S2 Appendix — (PDF) [file pone.0161477.s002.pdf]

Myopic Loss Aversion under Ambiguity and Gender Effects. S2 Appendix: Experimental Instructions (translated from Spanish)

Iñigo Iturbe-Ormaetxe [1], Giovanni Ponti[1,2,3,*], Josefa Tomás[1]

1. Departamento de Fundamentos del Análisis Económico, Universidad de Alicante, 03071 Alicante, Spain.
2. Department of Economics, University of Chicago, 1126 East 59th Street, Chicago IL 60637, United States.
3. Dipartimento di Economia e Finanza, LUISS Guido Carli, viale Romania 32, 00197, Roma, Italy.

* Corresponding Author. E-mail: giuba@ua.es.

Treatment 1 (T1, HF-R)

Welcome to this experiment in decision-making. The experiment will take about 30 minutes. Instructions are very simple and you can earn a sizable amount of money. The money that you earn will be paid in cash at the end of the experiment.

The experiment has nine rounds. In each round you will be endowed with 100 cents (€1). You will be asked to choose the portion x of this amount that you wish to invest in a risky option. This option is the same for the nine rounds.

The risky option: there is a 1/3 chance that the investment will be successful. If it is successful you will receive 2.5 times the amount you chose to bet. If the investment is unsuccessful (a chance of 2/3) you lose the amount you bet.

How do we determine if the investment is successful?

Each one of you has a record sheet with a letter A, B, or C at the top. In each round, one of you will take out a ball from a bag that contains three balls marked with the letters A, B, or C. If the letter chosen coincides with your letter you will win. Otherwise you will lose.

How can I calculate my earnings?

For each round, if you win you have to add your invested amount (x) multiplied by 2.5 to the initial endowment of 100 cents. If you lose, you have to deduct the invested amount (x) from the initial endowment.

At the end of the experiment you have to add up the amounts of the nine rounds and this is the total amount you will be paid in cash.

Treatment 2 (T2, LF-R)

Welcome to this experiment in decision-making. The experiment will take about 30 minutes. Instructions are very simple and you can earn a sizable amount of money. The money that you earn will be paid in cash at the end of the experiment.

The experiment has nine rounds divided in blocks of three rounds. In each round you will be endowed with 100 cents (€1). At the beginning of the first round you will be asked to choose the portion x of this amount that you wish to invest in the first,

second and third round. In the fourth round you have to choose the amount you wish to invest in the fourth, fifth and sixth rounds. Lastly, in the seventh round, you have to choose the amount for the seventh, eight and ninth rounds.
The risky option: there is a 1/3 chance that the investment will be successful. If it is successful you will receive 2.5 times the amount you chose to bet. If the investment is unsuccessful (a chance of 2/3) you lose the amount you bet.
How do we determine if the investment is successful?

Each one of you has a record sheet with a letter A, B, or C at the top. Every three rounds, at the end of the third, sixth and ninth rounds one of you will take out three balls from three bags, one from each bag. Each bag contains three balls marked with the letters A, B, or C.

If the letter taken out from the first bag coincides with the letter in your record sheet you win in the first round, otherwise you lose the invested amount. If the letter taken out from the second bag coincides with the letter marked in your record sheet you win in the second round and if the letter taken out from the third bag coincides with your letter you win in the third round.
How can I calculate my earnings?

After everybody has chosen the amount to bet in the first three rounds, the three balls will be taken out. In each round, if you win you have to add your invested amount (x) multiplied by 2.5 to the initial endowment of 100 cents. If you lose, you have to deduct the invested amount (x) from the initial endowment (100 cents). We will repeat this process for rounds 4 to 6 and then for rounds 7 to 9. Take note of the three partial results.

At the end of the three blocks you have to add up the partial amounts of the three blocks and this is the total amount you will be paid in cash.

Treatment 3 (T3, HF-A)

Welcome to this experiment in decision-making. The experiment will take about 30 minutes. Instructions are very simple and you can earn a sizable amount of money. The money that you earn will be paid in cash at the end of the experiment.

The experiment has nine rounds. In each round you will be endowed with 100 cents (€1). You will be asked to choose the portion x of this amount that you wish to invest in a risky option. This option is the same for the nine rounds.
The risky option: if your investment is successful you will receive 2.5 times the amount you chose to bet. If the investment is unsuccessful you lose the amount you bet.
How do we determine if the investment is successful?

Each one of you has a record sheet with a letter A, B, or C at the top. In each round, one of you will take out a ball from a bag that contains balls marked with the letters A, B, or C. The proportion of each letter is unknown. If the letter chosen coincides with your letter you will win. Otherwise you will lose.
How can I calculate my earnings?

For each round, if you win you have to add your invested amount (x) multiplied by 2.5 to the initial endowment of 100 cents. If you lose, you have to deduct the invested amount (x) from the initial endowment.

At the end of the experiment you have to add up the amounts of the nine rounds and this is the total amount you will be paid in cash.

Treatment 4 (T4, LF-A)

Welcome to this experiment in decision-making. The experiment will take about 30 minutes. Instructions are very simple and you can earn a sizable amount of money. The money that you earn will be paid in cash at the end of the experiment.

The experiment has nine rounds divided in blocks of three rounds. In each round you will be endowed with 100 cents (€1). At the beginning of the first round you will be asked to choose the portion x of this amount that you wish to invest in the first, second and third round. In the fourth round you have to choose the amount you wish to invest in the fourth, fifth and sixth rounds. Lastly, in the seventh round, you have to choose the amount for the seventh, eight and ninth rounds.

The risky option: if your investment is successful you will receive 2.5 times the amount you chose to bet. If the investment is unsuccessful you lose the amount you bet.

How do we determine if the investment is successful?

Each one of you has a record sheet with a letter A, B, or C at the top. Every three rounds, at the end of the third, sixth and ninth rounds one of you will take out three balls from three bags, one from each bag. Each bag contains balls marked with the letters A, B, or C. The proportion of each letter is unknown.

If the letter taken out from the first bag coincides with the letter in your record sheet you win in the first round, otherwise you lose the invested amount. If the letter taken out from the second bag coincides with the letter marked in your record sheet you win in the second round and if the letter taken out from the third bag coincides with your letter you win in the third round.

How can I calculate my earnings?

After everybody has chosen the amount to bet in the first three rounds, the three balls will be taken out. In each round, if you win you have to add your invested amount (x) multiplied by 2.5 to the initial endowment of 100 cents. If you lose, you have to deduct the invested amount (x) from the initial endowment (100 cents). We will repeat this process for rounds 4 to 6 and then for rounds 7 to 9. Take note of the three partial results.

At the end of the three blocks you have to add up the partial amounts of the three blocks and this is the total amount you will be paid in cash.
