# Supplementary material for: Myopic Loss Aversion under Ambiguity and Gender Effects
Source: PLoS One. 2016 Dec 14;11(12):e0161477. doi: 10.1371/journal.pone.0161477 (PMC5156393; doi:10.1371/journal.pone.0161477)
Supplement: S1 Table — (PDF) [file pone.0161477.s003.pdf]

Tab S3: Random-effect tobit regressions.

Vars.	Model 1	Model 2	Model 3
**LF**	27.79**	21.83*	50.65***
	(10.93)	(11.23)	(19.03)
**Uncert**	-13.28	-15.20	-28.07**
	(9.876)	(9.859)	(11.73)
**LF*Uncert**	1.180	3.271	-22.21
	(14.52)	(14.77)	(22.58)
**Female**	-0.557	0.401	-1.276
	(9.589)	(9.568)	(11.77)
**LF*Female**	-17.72	-15.63	-29.39
	(14.33)	(14.58)	(21.92)
**Uncert*Female**	-2.146	-1.107	6.268
	(13.37)	(13.32)	(15.70)
**LF*Uncert*Female**	10.26	10.24	17.42
	(20.10)	(20.08)	(22.48)
**Lastperiod**		13.56	30.07*
		(9.334)	(16.76)
**LF* Lastperiod**		7.034	29.37
		(11.77)	(24.83)
**Uncert* Lastperiod**		13.70	-3.791
		(11.08)	(17.55)
**LF* Uncert* Lastperiod**		-15.97	-29.64*
		(12.74)	(17.27)
**Loser**			0.561
			(7.431)
**LF* Loser**			-30.15
			(18.80)
**Uncert* Loser**			29.43***
			(9.286)
**LF* Uncert* Loser**			18.07
			(21.39)
**Female*Lastperiod**		-11.76	-14.13
		(10.77)	(16.60)
**LF* Female*Lastperiod**		1.229	5.091
		(12.76)	(14.53)
**Female*Loser**			5.597
			(9.340)
**LF* Female*Loser**			12.35
			(19.35)
**Lastperiod*Loser**			-18.73
			(17.81)
**LF* Lastperiod*Loser**			-17.07
			(20.70)
**Uncert*Female*Lastperiod**		-0.991	0.0128
		(12.70)	(14.42)
**Uncert*Female*Loser**			-17.80
			(11.99)
**Uncert*Lastperiod*Loser**			24.12
			(19.43)
**Female*Lastperiod*Loser**			-3.874
			(16.39)
